# Visual Quality and Subjective Satisfaction in Ultrathin Descemet Stripping Automated Endothelial Keratoplasty (UT-DSAEK) versus Descemet Membrane Endothelial Keratoplasty (DMEK): A Fellow-Eye Comparison

**DOI:** 10.3390/jcm10030419

**Published:** 2021-01-22

**Authors:** Josep Torras-Sanvicens, Irene Blanco-Domínguez, José-María Sánchez-González, Rahul Rachwani-Anil, Juan-Felipe Spencer, Noelia Sabater-Cruz, Jorge Peraza-Nieves, Carlos Rocha-de-Lossada

**Affiliations:** 1 Anterior Segment Department, Institut Clinic d’Oftalmologia, Hospital Clinic of Barcelona, 08036 Barcelona, Spain; jts29206@gmail.com (J.T.-S.); ire_blanco@hotmail.com (I.B.-D.); felipe.spencerv@gmail.com (J.-F.S.); noelia.sabater@gmail.com (N.S.-C.); jorge.peraza.nieves@gmail.com (J.P.-N.); carlosrochadelossada5@gmail.com (C.R.-d.-L.); 2 Ophthalmology Department, University of Barcelona, 08007 Barcelona, Spain; 3 Physics of Condensed Matter Department, Optics Area, University of Seville, 41012 Seville, Spain; 4 Ophthalmology Department, Regional Universitary Hospital of Malaga, 29010 Malaga, Spain; rahul.medum@gmail.com; 5 Ophthalmology Department, University of Malaga, 29016 Malaga, Spain; 6 Ophthalmology Department, Virgen de las Nieves Hospital, 18014 Granada, Spain

**Keywords:** ultra-thin DSAEK, DMEK, endothelial keratoplasty, objective visual quality, subjective visual quality, Fuchs endothelial dystrophy

## Abstract

Background: To analyze objective and subjective visual quality differences between descemet membrane endothelial keratoplasty (DMEK) and ultra-thin descemet stripping automated endothelial keratoplasty (UT-DSAEK) with a paired contralateral-eye design. Methods: A cross-sectional, comparative, and observational case series study between DMEK and UT-DSAEK were presented. Visual acuity, refractive status and corneal quality assessment were compared between both endothelial keratoplasty techniques. The sample consisted of 20 eyes (10 patients) diagnosed with Fuchs endothelial corneal dystrophy. All measurements were performed preoperatively and at six months after surgery. Analyzed data included the measurement of objective scattering index, modulation transfer function, Strehl ratio, and optical quality assessment (OQAS) values. Contrast sensitivity, subjective patient satisfaction, visual acuity, tomography, pachymetry, endothelial cell count, and refraction status were also analyzed. Results: Objective and subjective visual quality variables had similar results among UT-DSAEK and DMEK procedures. Statistically significant differences favoring DMEK against UT-DSAEK were found in endothelial cell density (658.80 ± 139.33 and 1059.00 ± 421.84 cells/mm^2^, respectively), pachymetry (621.20 ± 33.74 and 529.70 ± 30.00 µm, respectively), and follow-up (45.50 ± 24.76 and 15.50 ± 8.43 months, respectively). Conclusions: UT-DSAEK and DMEK revealed no differences in terms of objective and subjective visual quality. However, DMEK showed a faster recovery during the follow-up, increased endothelial cell density, lower pachymetry, and a more anatomical posterior keratometry against UT-DSAEK in this case series paired-eye study.

## 1. Introduction

Endothelial lamellar keratoplasty was popularized twenty years ago, completely displacing penetrating keratoplasty (PK) as the gold-standard treatment for endothelial disorders [1]. Within the current endothelial keratoplasty procedures, Descemet stripping automated endothelial keratoplasty (DSAEK) and non-automatic (DSEK) were first described, involving a healthy donor graft consisting of endothelium and a section of the posterior stroma. DSAEK is actually on the most performed endothelial transplant techniques in the United States of America [2]. The DSAEK technique has been partially replaced by Descemet membrane endothelial keratoplasty (DMEK), as described by Melles [3], in which only the endothelium and the Descemet membrane (DM) are grafted. DMEK represents a greater technical challenge, since the graft fragility makes it more difficult to separate from the donor eye as well as to deploy the graft onto the recipient cornea. Moreover, there is a greater risk of graft detachment and primary failure in the early postoperative period [4]. Eye banks have minimized the preparation risk, yet many surgeons prefer to avoid the stated intra and postoperative DMEK challenges [5].

Ultrathin DSAEK (UT-DSAEK) is defined when the graft thickness is less than 100 µm, and it has demonstrated superior visual acuity than conventional DSAEK (above 150 µm) [6]. Recently, Chamberlain et al. [4,7] reported a Descemet endothelial thickness comparison trial (DTCT), concluding that DMEK achieved better visual acuity and fewer higher order aberrations (HOA) at three, six, and twelve months after surgery compared to UT-DSAEK. However, Dunker et al. [8] reported no statistically significant differences in visual acuity outcomes between DMEK and UT-DSAEK. Some recent studies were included in a systematic review by Stuart et al. [9] with a paired contralateral-eye design (DMEK versus DSAEK). The systematic review concluded that DMEK achieved better best corrected visual acuity (BCVA) and a faster visual recovery than DSAEK. Despite this evidence, DSAEK is still a very widespread technique, and some surgeons are reluctant to switch to the DMEK procedure. On the one hand, DMEK outcomes have proved to have a higher dislocation and a higher graft failure rate than DSAEK [9]. On the other hand, these events seem to be related to the surgeon’s experience [10]. Despite all of the evidence regarding satisfactory visual and refractive results in endothelial keratoplasties, currently little is known regarding visual quality among both techniques, as most of the research available has compared their visual acuity outcomes using different cohorts of patients (DMEK vs. DSAEK), and few variables based on visual quality have been taken into account [4,8].

The purpose of this study was to analyze objective and subjective visual quality differences between DMEK and UT-DSAEK with a paired contralateral-eye design case series study. In addition, visual acuity, refractive status, and corneal quality assessment were compared between both endothelial keratoplasty techniques.

## 2. Materials and Methods

### 2.1. Design

We presented a cross-sectional, comparative, and observational case series medical chart review study involving DMEK and UT-DSAEK outcomes. All 13 patients from our cohort (26 eyes) were diagnosed with Fuchs endothelial corneal dystrophy (FECD). In all patients, UT-DSAEK was performed in the first eye and DMEK in the second eye. The same ophthalmologist performed all surgeries. All individuals were recruited from Hospital Clinic, Barcelona. Surgical procedures were performed from February 2017 to February 2019 for DMEK and between April 2012 to February 2018 for UT-DSAEK. The exclusion criteria were (1) eyes with no follow-up data, (2) previous corneal surgery, (3) complicated intraoperative or postoperative course (difficulty or inability to unfold or center the graft, graft expulsion and subsequent over-manipulation, and postoperative complications such as air/gas pupillary block glaucoma with posterior atonic mydriasis or long term graft detachment with no complete edema resolution), and (4) vision-limiting ocular comorbidities. In addition, (5) bullous keratopathy, and (6) other causes of endothelial decompensation, such as prior intraocular surgery, trauma, glaucoma drainage devices, or existence of concomitant ocular pathology were excluded. Transparency of the IOL-posterior capsule complex was verified in all of them. Elsching pearls and significant fibrosis were ruled out with retro illumination after dilating the pupil. One patient was excluded by age-related macular degeneration in the UT-DSAEK group, and two patients by graft failure due to low ECD. Ethical approval was obtained by the Hospital Clinic of Barcelona Committee (HCB/2019/0570), and the study was conducted according to the tenets of the Declaration of Helsinki (seventh revision, Fortaleza, Brazil). All patients signed an institutional review board-approved informed consent form before surgery.

### 2.2. Surgical Procedures

#### 2.2.1. UT-DSAEK

All surgeries were performed using pre-cut donor corneas (from the Barcelona Eye Bank, Barcelona, Spain). Graft thickness in all cases was less than 100 µm, and grafts were stored in a specific organ culture medium (CorneaMax; Eurobio, Les Ulis Cedex, France). The pre-cut donor cornea was trephined at a diameter of 8.0 to 8.5 mm (Barron Corneal Punch 8.0 mm, Katena, Parsippany, NJ, USA), marked with an asymmetric letter on the stromal side, and placed into a glide (Busin glide, Moria SA, Antony, France). We carried out the “pull-through” technique described by Busin et al. [11]. After descemetorhexis under air, the graft was placed into the glide (Busin glide, Moria SA, Moria SA, Antony, France) and pulled using Busin forceps through the 4.00 mm main incision into the anterior chamber. Finally, the incisions were sutured with Nylon 10-0, and an air tamponade was carried out.

#### 2.2.2. DMEK

DMEK graft preparation was performed in the Barcelona Eye Bank with a non-touch technique. DM was partially lifted with a blunt curved McPherson forceps, laid back onto the posterior corneal surface maintaining a hinge of DM attached to the stroma, and sent to our hospital preserved in an organic culture medium (CorneaMax; Eurobio, Les Ulis Cedex, France). The day of the surgery, the DMEK graft was partially lifted with a blunt Troutman forceps and displayed like “a taco” to expose the posterior stroma. A stromal window was made trephining with a 2 mm dermatologic punch (BP-20F; Kai medical Europe, Solingen, Germany), the DM was placed again over the stroma, turned over, and marked with an asymmetric letter using the tip of a Sinskey hook through the 2 mm window. Using an 8.0- or 8.5-mm trephine (Barron Corneal Punch 8.0 mm, Katena, Parsippany, NJ, USA), the graft was cut and then separated from the stromal bed with Troutman forceps. Trypan blue 0.06% (Vision blue, DORC, Zuidland, The Netherlands) was applied for four minutes onto the graft for staining and then charged in a glass injector (Geuder AG, Heidelberg, Germany). DMEK surgery was performed, removing the recipient’s DM under air with a blunt reverse Sinskey hook (descemetorhexis). Later, the DMEK graft was unfolded, the correct orientation was assured (with the visualization of the asymmetric letter previously marked), and the graft was centered and lifted against the recipient posterior stroma by injecting an air bubble into the anterior chamber.

### 2.3. Measurements

#### 2.3.1. Objective and Subjective Visual Quality

Objective visual quality was performed with an Optical Quality Analysis System (OQAS; Visiometrics SL, Terrassa, Spain). The following measurements were carried out: (I) Objective scattering index (OSI): an objective evaluation of intraocular scattered light. This index is calculated by evaluating the amount of light outside the double-pass retinal intensity point spread function (PSF) image in relation to the amount of light in the center. (II) Modulation transfer function (MTF) cutoff value: frequency at which the MTF reaches a value of 0.01. It refers to the spatial frequency, perceived by the eye, with a significant 1% contrast. (III) the Strehl ratio (SR): ratio of the central maximum of the PSF illuminance in the aberrated eye divided by the central maximum that would be expected in a corresponding aberration-free system. (IV) The OQAS values (OVs): The three OVs are normalized values of three spatial frequencies, which correspond to MTF values that describe the optical quality of the eye for three different contrast conditions commonly used in ophthalmic practice: 100% (OV 100%), 20% (OV 20%), and 9% (OV 9%). Contrast sensitivity was measured using a Pelli–Robson chart. Subjective patient satisfaction was analyzed with the questionnaire published by Goldich et al. [12]. This questionnaire is the only currently available tool suitable for fellow-eye comparison studies.

#### 2.3.2. Visual Acuity and Refraction Status

Patients were routinely examined at the preoperative and postoperative visit. Best corrected distance visual acuity (BCDVA), uncorrected distance visual acuity (UDVA), and corrected distance visual acuity (CDVA) were measured using a 20-feet Snellen chart in photopic conditions and converted to the logarithm of the minimum angle of resolution (logMAR) for statistical analysis. Keratometry, higher-order, and total aberration measurements were assessed by Scheimpflug imaging software (Pentacam HR; Oculus, Wetzlar, Germany). The device emits a blue light beam (475 nm wavelength) and simultaneously, an integrated camera measures 25,000 points and reconstructs a series of 25 images (1003 × 520 pixels) of different corneal meridians.

For the densitometry analysis, the program automatically locates the corneal apex and measures the density of the cornea divided in two concentric zones (central zone: 2 mm; first ring: 2–6 mm) and in 3 different layers in depth (anterior: 120 µm; posterior: 60 µm; and central: thickness between anterior and posterior). Corneal densitometry is expressed in gray scale units (GSU) in a range from 0 to 100, where 0 means minimum light backscattering and hence maximum transparency, and 100 means maximum backscattering and therefore no transparency. Total corneal thickness and corrected corneal thickness were measured with the caliper system of DRI Triton Swept-Source Ocular Coherence Tomography (Topcon Medical System, Tokyo, Japan). The first point of the OCT caliper was placed on the interface corneal graft center, and the second point at the endothelium of the graft. Endothelial cell density (ECD) assessment was performed with a Corneal Specular Microscope SP (Topcon Medical Systems, Tokyo, Japan). ECD was an automatic cell count procedure. The ECD image was taken of the central cornea. All measurements were performed at six months after surgery.

### 2.4. Statistical Analysis

Data were analyzed with SPSS statistics software (version 26.0 for Windows; SPSS Inc., Chicago, IL, USA). Descriptive analysis was carried out with values expressed in mean ± SD. Data normality distribution was assessed with the Shapiro–Wilk test for DMEK and UT-DSAEK groups. Differences in mean values between both eyes was assessed with “U” in the Mann–Whitney test and chi square test. Effect size calculation was assessed by the “D” of Cohen’s test [13]. Power calculation was assessed with the GRANMO calculator (Institut Municipal d’Investigació Mèdica, Barcelona, Spain; Version 7.12). Accepting an alpha risk of 12:05 in a two-sided test with 10 subjects in the first group and 10 in the second, the statistical power was 90% to recognize as statistically significant a difference of ECD means (658.8 in group 1 and 1059 in group 2). For all tests, the level of significance was established at 95% (*p* < 0.05).

## 3. Results

Twenty eyes from 10 patients were included in this cross-sectional study. Four patients were male and six were female. Six right eyes and four left eyes were UT-DSAEK, while four right eyes and six left eyes were DMEK. All patients had previous IOL surgery. Patients’ mean age was 75.40 ± 6.69 years. UDVA, CDVA, refraction, ECD, follow-up, rebubbling, pachymetry, HOA, mean keratometry, and corneal densitometry differences between UT-DSAEK and DMEK surgeries are presented in Table 1. Mean UDVA was 0.49 ± 0.22 LogMAR in the UT-DSAEK group and 0.43 ± 0.17 LogMAR in the DMEK group (*p* = 0.68). Previous total pachymetry was 671.12 ± 60.90 µm in the UT-DSAEK arm and 656.28 ± 39.42 µm in the DMEK arm (*p* = 0.77). Preoperative graft eye bank ECD was 2520.00 ± 257.33 (2200 to 3100) in the UT-DSAEK group and 2670.00 ± 194.65 (2400 to 2900) in the DMEK group (*p* = 0.14). Therefore, neither variables showed statistically significant differences, and both groups were comparable at baseline.

Statistically significant differences in favor of DMEK surgery were found in ECD, follow-up, total pachymetry, and posterior mean keratometry. Preoperative versus postoperative surgery ECD achieved statistically significant differences in both procedures (*p* < 0.01) Pachymetry values after subtracting graft thickness (in UT-DSAEK group measured by OCT) were almost identical between fellow eyes (*p* = 0.92). Effect size for statistically significant differences was 1.27 for ECD, 1.63 for follow-up, 2.86 for total pachymetry, and 1.59 for posterior keratometry. All effect size values represented a large difference according to “D” of Cohen’s test.

In addition, objective visual quality variables (OSI, MTF, Strehl Ratio, OV100%, OV20%, OV9%, and contrast sensitivity) and subjective visual quality variables (subjective quality of the surgical technique, level of comfort in the postoperative period, recovery time, and preferred eye) are presented in Table 2. No variable achieved statistically significant differences when comparing UT-DSAEK and DMEK procedures. Within the complications, we found one re-UT-DSAEK and one re-DMEK due to early graft failure. Furthermore, one patient presented partial opacification of a hydrophilic intraocular lens (IOL) in both eyes, and another patient experienced a pupillary block after undergoing a UT-DSAEK procedure.

## 4. Discussion

Corneal transplantation has evolved in recent years with endothelial transplants currently being the selection for endothelial damage, surpassing PK. Although DMEK seems to offer better and faster visual and refractive results, controversy still exists till today on whether it is a better technique than DSAEK and especially than UT-DSAEK, due to the latter being a relatively easier technique and with similar outcomes to DMEK [8]. Dickman et al. [6] compared DSAEK with UT-DSAEK in a double masked randomized clinical trial (RCT) and found faster and better recovery of BCVA in the last one, with similar refractive outcomes, endothelial cell loss, and incidence of complications. Furthermore, little is known regarding patient preferences, reported outcomes, and objective visual quality variables between both surgeries. We observed that there were no statistically significant differences related to both objective and subjective visual qualities between DMEK and UT-DSAEK using the paired eye as a control.

Regarding ECD, Guerra et al. [10] found a similar loss at one year postoperative between both procedures. At the 6- and 12-month postoperative examinations, the mean BCVA was significantly better in the DMEK eyes (0.08 logMAR (20/24) and 0.07 logMAR (20/23), respectively) than in the DSAEK group (0.19 logMAR (20/31) and 0.20 logMAR (20/32), respectively), with *p* values of 0.005 and 0.004, respectively, reaching a greater percentage of patients with 20/25 and/or 20/20 in DMEK versus DSAEK. Regarding patient’s preference, 85% preferred the eye treated with DMEK when asked which eye had better quality of vision. Unlike our study, Guerra et al. [10] compared DSAEK, not UT-DSAEK, and they did not measure objective quality of vision items but only subjective items through questionnaires. Goldich et al. [14] reported similar findings to us, in a paired eyed study, observing that eyes after DSAEK surgery had statistically significantly thicker corneas and steeper posterior corneal curvatures compared to the fellow eyes that underwent DMEK. This is in line with our results, as we observed a steeper posterior cornea in the UT-DSAEK group (−6.98 ± 0.30) compared to the DMEK group (−6.45 ± 0.36). Likewise, Goldich found that posterior curvature was flatter in eyes with DMEK compared to eyes that underwent DSAEK, with statistically significant differences. Moreover, our results also agreed that anterior mean keratometry did not show statistically significantly differences among both procedures.

Goldich et al. [12], in a second study using the paired eye as a control, found a statistically significantly better BCVA in the DMEK group than in the DSAEK group (0.25 ± 0.1 logMAR and 0.39 ± 0.1 logMAR, respectively (*p* = 0.02)) at the 6-month visit. Regarding ECD loss and similar to our results, they observed a higher ECD loss in the DSAEK group (35.6%) during the first 6 months compared to the DMEK group (15.8%). Regarding visual outcomes, unlike our patients, most of their patients preferred DMEK surgery. Moreover, they reported better subjective visual outcomes and higher satisfaction rates in the DMEK group. As opposed to our study, they used DSAEK instead of UT-DSAEK. This fact may explain the differences observed between the findings of both studies. Interestingly, a statistically non-significant difference was found regarding time to resume daily activities after surgery. Goldich et al. [12] reported thicker corneas in DSAEK (627.9 ± 70 microns) vs. DMEK (541.0 ± 61). They did not report if the graft was considered, but we assume that it was. Similarly, our results are in the same direction where the DSAEK groups, considering the thickness of the graft, have thicker corneas (530.10 ± 29.89 µm) than the DMEK groups (529.70 ± 30.00 µm, *p* = 0.92). When we eliminated the thickness of the graft, and we only measured the thickness of the cornea, we could observe that there were no statistically significant differences in the corneal thickness between both techniques.

Bhandari et al. [15] reported that eyes undergoing DMEK had a statistically better improvement in visual acuity than the fellow eyes undergoing DSAEK (*p* < 0.05). Unlike our and other author’s results [12], they found no statistically significant differences (*p* = 0.08) in the ECD loss between the two groups at one year after the surgery. Maier et al. [16], in a study with ten patients, observed a significantly higher BCVA in the DMEK arm compared to the DSAEK arm (0.16 ± 0.10 vs. 0.45 ± 0.58 logMAR, *p* = 0.043). Unlike our results, contrast threshold, in their case using isolated Landolt ring, was significantly higher after DMEK than after DSAEK (0.49 ± 0.23 vs. 0.25 ± 0.18, *p* ± 0.043). However, similar to our findings, post-surgery astigmatism, mean spherical equivalent, and HOA did not differ. In line with other research, most of their patients preferred the DMEK procedure.

Regarding the subjective data, according to the adapted questionnaire from Goldich et al. [14], our case series showed non-significant differences concerning visual recovery, although in favor of DMEK. Six patients preferred DMEK, three patients reported in favor of UT-DSAEK, and one patient did not report any preference. In addition, DSAEK preference patients experienced higher rebubbling incidence in the fellow DMEK eye. Non statistically significant differences were found regarding corneal densitometry between UT-DSAEK and DMEK.

It is known that HOAs may increase after endothelial keratoplasty, which would significantly reduce visual acuity presumably due to the degradation of the retinal image point scattering function, and may have a greater impact than the induced light scattering [17]. Recently, Duggan et al. [18] reported in an RCT that DMEK resulted in less posterior corneal HOA compared to UT-DSAEK, suggesting that the total posterior corneal HOA correlates moderately with 6- and 12-month post-operative visual acuity and may partially account for the better visual acuity observed after DMEK. In this study, we did not observe statistically significant differences in total, anterior, or posterior HOAs; however, our sample size was limited, as it was a paired-eye study. Moreover, while observing the posterior HOAs between groups, we found less posterior HOA in the DMEK group with a *p* value of 0.07, hence assuming that with a larger sample we could reach statistically significant differences. Like Duggan et al. [18], HOA of the anterior corneal surface in our sample did not differ significantly between DMEK and UT-DSAEK.

Mencucci et al. [19] compared UT-DSAEK and DMEK in a similar study to ours, finding a difference of 0.023 logMAR favoring DMEK in BCVA. Moreover, clinical significant differences (2.5 letters or more) were considered unlikely among both procedures. However, regarding total and posterior corneal higher order aberrations (HOAs), posterior astigmatism and total coma were significantly lower after DMEK than UT-DSAEK at both 4- and 6-mm optical zones. Additionally, they found that contrast sensitivity was higher after DMEK, especially in mesopic conditions and at medium spatial frequencies. Menucci et al. [19] observed that the ECD was higher in the UT-DSAEK group; however, they did not observe statistically significant differences between both techniques. Regarding patient preference, they reported that although most patients were highly satisfied with their vision in both eyes, about one third reported better vision in the eye that underwent DMEK.

Recently, the DETECT study [4], a double masked RCT, confirmed a better visual outcome without increasing surgical complications in eyes randomized for DMEK versus the eyes randomized for UT-DSAEK. However, other authors have reported similar visual results or little evidence of superiority in favor of DMEK, especially when compared with UT-DSAEK grafts less than 100 µm of thickness [20]. In the same line, Dunker et al. [8] reported in another RCT no significant differences regarding visual acuity, although a higher percentage of patients attained 20/25 in the DMEK arm (66% of 29 eyes) compared to the DSAEK arm (33% of 25 eyes) at 12 months of follow-up. Interestingly, ECD loss did not differ significantly between both treatment groups. Moreover, a second analysis of the DETECT study found that improvements in vision-related quality of life did not prove to be greater in the DMEK arm compared to the UT-DSAEK arm [21].

To the best of our knowledge and to date, this is the only paired study with contralateral eyes in which the objective visual quality of the eyes treated with DMEK and UT-DSAEK has been studied using the Optical Quality Analyzer (OQAS) with a double-pass system of a light beam that obtains a value from the scattering of a point of light throughout the optical system. No significant differences were found in any of the parameters studied, including OSI, MTF-cut off, and Strehl ratio, although values did vary among patients. Other non-corneal optical aspects that could have influenced light scattering, such as the lens and the bag-IOL complex, were ruled out. In this regard, all cases were pseudophakic at the time of measurement, and the transparency of the IOL-posterior capsule complex was verified in all of them except for one patient with partial opacification of a hydrophilic IOL in both eyes, yet respecting the visual axis in both eyes.

Among the limitations of our study, this is a retrospective-cross sectional serial case with a low number of patients. However, there are very few studies reporting the outcomes of DMEK vs. UT-DSAEK with the paired eye being used as a control. Moreover, as far as we know, we are the first to report the objective and subjective visual quality differences between DMEK and UT-DSAEK with a paired contralateral-eye design using the Optical Quality Analyzer (OQAS) with a double-pass system. We cannot ignore any possible bias, since the patients reported their outcomes in a cross-sectional way. Our study, like the other comparative studies of contralateral eyes, presents several biases. The most important bias is that the eye operated in the first place was always UT-DSAEK, and the second eye undergoing DMEK was performed knowing the result of the first eye. Another confounding factor is the fact that two eyes in the UT-DSAEK group and no patient in the DMEK group underwent cataract surgery at the same time. Nevertheless, we do not believe that this influences the quality of the graft that was inserted after cataract surgery and after the ocular viscoelastic device was totally removed. Moreover, our study is in line with Goldich et al. [12], where nine patients in the DMEK group and ten in the DSAEK group underwent phacoemulsification at the same time. As new lines of research emerge with larger sample sizes, the study and analysis of corneal density patterns will be carried out.

## 5. Conclusions

According to our results, UT-DSAEK and DMEK procedures showed no differences in terms of objective and subjective visual quality. DMEK outcomes showed a better recovery during follow-up, higher ECD, lower pachymetry, and a flatter posterior keratometry compared to UT-DSAEK outcomes in this case series paired-eye study.

## Figures and Tables

**Table 1:** Postoperative visual acuity, and refraction and tomograph differences between UT-DSAEK and DMEK.

Variable (Units)	UT-DSAEK (*n* = 10)	DMEK (*n* = 10)	*p* Value
UDVA (LogMAR)	0.56 ± 0.27 (1.00 to 0.30)	0.58 ± 0.33 (1.00 to 0.10)	0.73
CDVA (LogMAR)	0.16 ± 0.14 (0.50 to 0.00)	0.21 ± 0.29 (1.00 to 0.00)	0.99
Spherical equivalent (diopters)	−0.42 ± 1.52 (−3.50 to +1.75)	−0.47 ± 1.16 (−2.50 to +1.25)	0.85
Cylinder refraction (diopters)	−2.20 ± 1.39 (−5.00 to −0.75)	−1.72 ± 0.87 (−3.00 to −0.50)	0.48
Endothelial cell density (cells/mm^2^)	658.80 ± 139.33 (462.00 to 931.00)	1059.00 ± 421.84 (452.00 to 1941.00)	<0.05
Follow-up (latest appointment) (months)	45.50 ± 24.76 (15 to 90)	15.20 ± 8.43 (4.00 to 28.00)	<0.01
Rebubblings (number)	0.40 ± 0.69 (0.00 to 2.00)	0.60 ± 0.96 (0.00 to 3.00)	0.73
Tomograph Measurements			
Total pachymetry (µm)	621.20 ± 33.74 (571.00 to 677.00)	529.70 ± 30.00 (496.00 to 576.00)	<0.01
Graft pachymetry (µm)	91.10 ± 25.24 (45.00 to 134.00)	-	-
Pachymetry without graft (µm)	530.10 ± 29.89 (503.00 to 577.00)	529.70 ± 30.00 (496.00 to 576.00)	0.92
Anterior HOA	0.27 ± 0.15 (0.11 to 0.65)	0.38 ± 0.22 (0.14 to 0.97)	0.12
Posterior HOA	0.25 ± 0.08 (0.17 to 0.42)	0.18 ± 0.09 (0.08 to 0.37)	0.07
Total HOA	0.37 ± 0.19 (0.13 to 0.73)	0.43 ± 0.22 (0.20 to 0.98)	0.68
Anterior mean keratometry (diopters)	42.94 ± 1.80 (40.60 to 46.00)	42.96 ± 1.64 (40.40 to 46.20)	0.97
Posterior mean keratometry (diopters)	−6.98 ± 0.30 (−7.30 to −6.40)	−6.45 ± 0.36 (−6.90 to −5.80)	<0.01
Corneal Densitometry (GSU)			
Anterior (0.00 to 2.00 mm)	31.39 ± 6.45 (23.00 to 43.60)	28.71 ± 4.48 (20.70 to 36.80)	0.35
Central (0.00 to 2.00 mm)	20.05 ± 3.14 (16.50 to 25.20)	19.52 ± 2.73 (15.70 to 25.00)	0.68
Posterior (0.00 to 2.00 mm)	18.48 ± 4.26 (14.50 to 28.30)	15.75 ± 2.63 (11.30 to 20.80)	0.16
Anterior (2.00 to 6.00 mm)	32.52 ± 6.93 (21.30 to 41.70)	28.56 ± 4.14 (19.70 to 36.50)	0.31
Central (2.00 to 6.00 mm)	21.23 ± 3.99 (15.20 to 29.10)	19.89 ± 3.13 (14.90 to 25.70)	0.52
Posterior (2.00 to 6.00 mm)	20.03 ± 5.77 (13.90 to 31.50)	16.54 ± 2.82 (11.50 to 21.20)	0.35

Measurements were expressed as mean ± standard deviation (range). UDVA: uncorrected distance visual acuity (LogMAR), CDVA: corrected distance visual acuity (LogMAR), HOA: high order aberrations, GSU: gray scale units.

**Table 2:** Postoperative objective and subjective visual quality differences between UT-DSAEK and DMEK.

Variable (Units)	UT-DSAEK (*n* = 10)	DMEK (*n* = 10)	*p* Value
Objective Visual Quality			
Objective scattering index	4.58 ± 3.20 (1.50 to 11.50)	4.14 ± 3.47 (0.90 to 11.70)	0.68
Modulation transfer function	12.77 ± 5.80 (4.35 to 19.39)	16.95 ± 9.57 (2.77 to 32.78)	0.43
Strehl ratio	0.08 ± 0.02 (0.05 to 0.12)	0.10 ± 0.04 (0.04 to 0.17)	0.28
OQAS value 100% (LogMAR)	0.45 ± 0.26 (1.00 to 0.20)	0.33 ± 0.31 (1.00 to 0.00)	0.28
OQAS value 20% (LogMAR)	0.61 ± 0.27 (1.00 to 0.40)	0.49 ± 0.29 (1.00 to 0.20)	0.43
OQAS value 9% (LogMAR)	0.73 ± 0.20 (1.00 to 0.50)	0.65 ± 0.22 (1.00 to 0.40)	0.39
Contrast sensitivity	1.48 ± 0.19 (1.05 to 1.65)	1.53 ± 0.15 (1.20 to 1.65)	0.63
Subjective Visual Quality			
Subjective quality of surgical technique (from 1 to 6)	4.70 ± 0.91 (3.00 to 6.00)	4.90 ± 0.61 (4.00 to 6.00)	0.79
Comfortability of postoperative period (from 1 to 6)	4.40 ± 1.34 (1.00 to 6.00)	4.60 ± 1.07 (3.00 to 6.00)	0.91
Recovery time (weeks)	7.40 ± 7.01 (2.00 to 24.00)	3.80 ± 1.03 (2.00 to 6.00)	0.16
Preferred eye * (Right or Left)	3	6	0.99

Measurements were expressed as mean ± standard deviation (range). OQAS: Optical Quality Analysis System. * One patient did not state the preferred eye.

## Data Availability

The data presented in this study are available on request from the corresponding author. The data are not publicly available due to the data are part of future studies in other lines of research.
